# Supplementary material for: Annihilation-limited long-range exciton transport in high-mobility conjugated copolymer films
Source: Proc Natl Acad Sci U S A. 2025 Apr 22;122(17):e2413850122. doi: 10.1073/pnas.2413850122 (PMC12054736; doi:10.1073/pnas.2413850122)
Supplement: Supplementary file 1 — Appendix 01 (PDF) [file pnas.2413850122.sapp.pdf]

PNAS

Supplementary Information Appendix for

Annihilation-limited Long-range Exciton Transport in High-mobility Conjugated Copolymer Films

Yuping Shi[a,b,c,*], Partha P. Roy[a,b,d], Naoki Higashitarumizu[e,f,g], Tsung-Yen Lee[a], Quanwei Li[a], Ali Javey[e,f], Katharina Landfester[c], Iain McCulloch[h,i], and Graham R. Fleming[a,b,*]

[a]Department of Chemistry, University of California, Berkeley, CA 94720, USA.

[b]Molecular Biophysics and Integrated Bioimaging Division, Lawrence Berkeley National Laboratory, Berkeley, CA 94720, USA.

[c]Max Planck Institute for Polymer Research, Mainz 55128, Germany.

[d]Department of Chemistry, Northwestern University, IL 60208, USA.

[e]Electrical Engineering and Computer Sciences, University of California, Berkeley, CA 94720, USA.

[f]Materials Sciences Division, Lawrence Berkeley National Laboratory, Berkeley, CA 94720, USA.

[g]JST, PRESTO, 4-1-8 Honcho, Kawaguchi, Saitama 332-0012, Japan.

[h]Department of Chemistry, University of Oxford, Oxford OX1 3TA, UK.

[i]Department of Electrical and Computer Engineering, Princeton University, New Jersey 08544, USA.

[*]Corresponding Authors. Email: shiy@mpip-mainz.mpg.de (Y.S.); grfleming@lbl.gov (G.R.F).

This appendix includes: Section I. **Fig. S1** to **S17**, and **Table S1**

Section II. Two-component PP5 Analysis

Section III. Photocurrent in PCBM/IDTBT Blend Thin Film

Section IV. List of Materials and Sources Used in Fig. 3

Section V. Systematic Error Evaluation

Section VI. Phenomenological EEA Model

<u>SECTION I</u>. Supplementary Data and Discussion

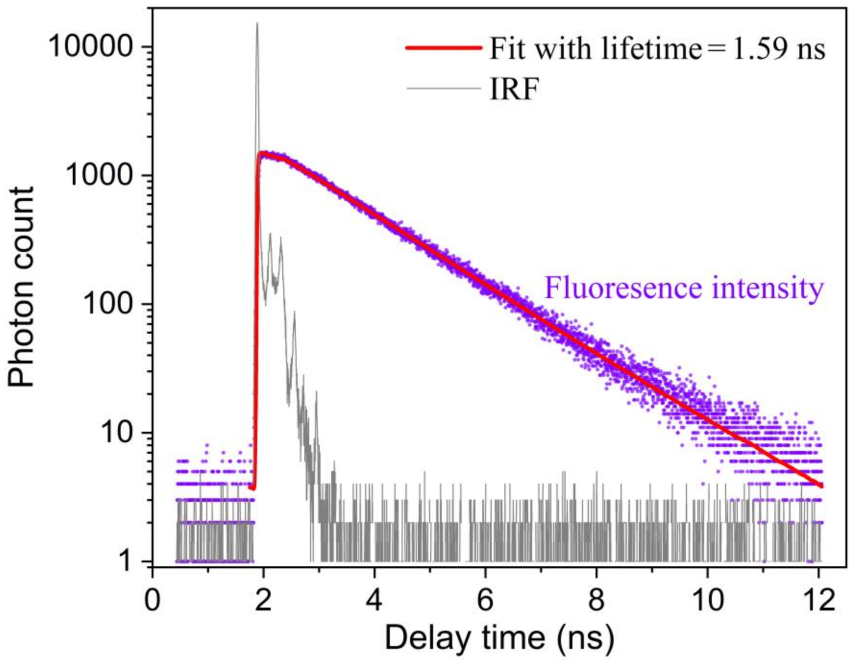

Fig. S1. Time-correlated single photon counting (TCSPC) fluorescence lifetime of IDTBT solution at room temperature. The IRF spectrum is the instrument response function signal of our TCSPC setup to the excitation laser.

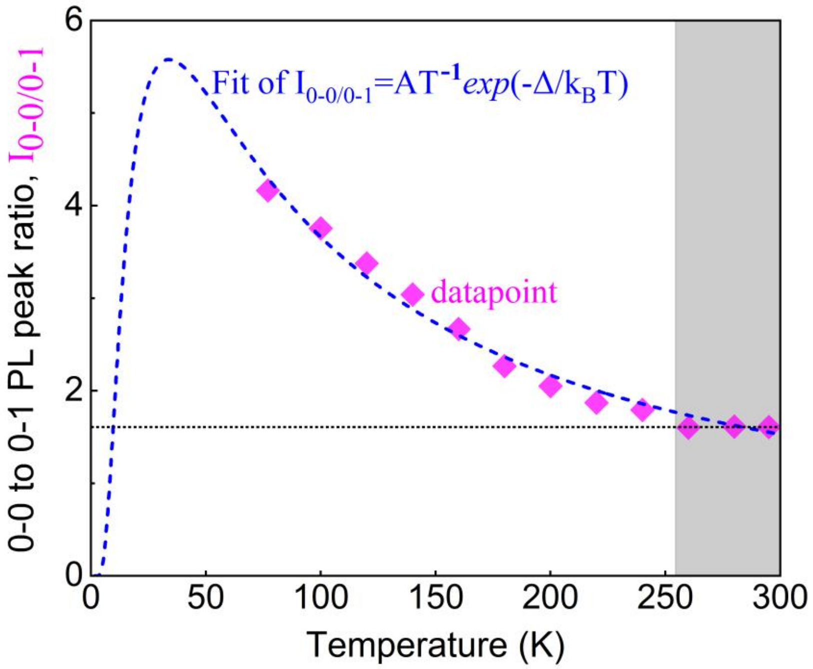

Fig. S2. Fit of $I_{0\text{-}0/0\text{-}1} = A \times T^{-1} \times \exp(-\Delta/k_B T)$ dependence as a function of temperature (T), with respect to the 0-0 to 0-1 PL vibronic peak ratio ($I_{0\text{-}0/0\text{-}1}$) data in a representative IDTBT thin-film sample. Herein, Δ denoting the interchain exciton bandwidth is estimated to be 23.6 ± 3.5 cm^{-1}, while $A \approx 514$ is a fitting amplitude and k_B the Boltzmann constant. The horizontal dotted black line delineates an average of the $I_{0\text{-}0/0\text{-}1}$ data obtained at 295 K, 280 K and 260 K, with the right grey-shaded rectangle highlighting this temperature range in which the $I_{0\text{-}0/0\text{-}1}$ data remain at the same level. An estimate of such low Δ may indicate the formation of extremely straight backbone conformations and also weak interchain coupling at chain-chain π-π crossing points (Ref. S1).

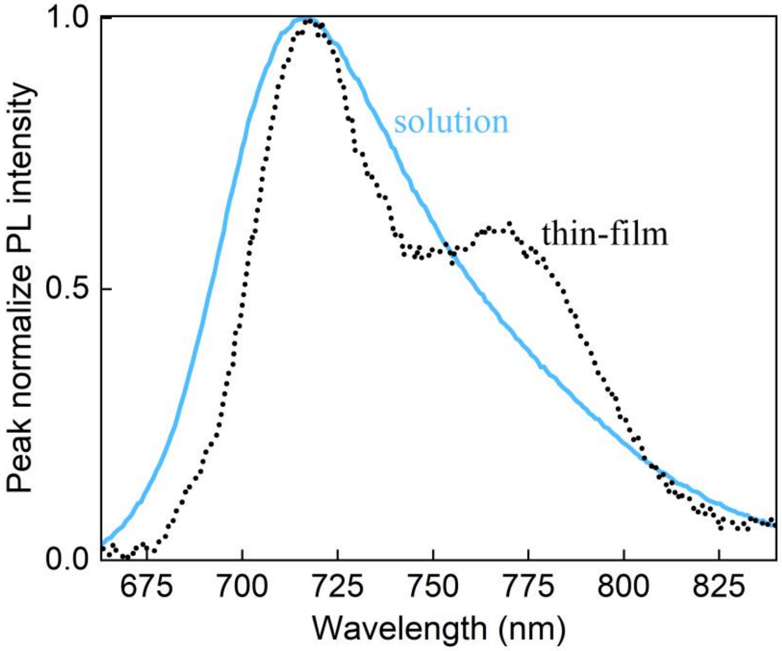

Fig. S3. Comparison of the peak-normalized steady-state photoluminescence (PL) spectra measured in IDTBT thin-film and solution at room temperature.

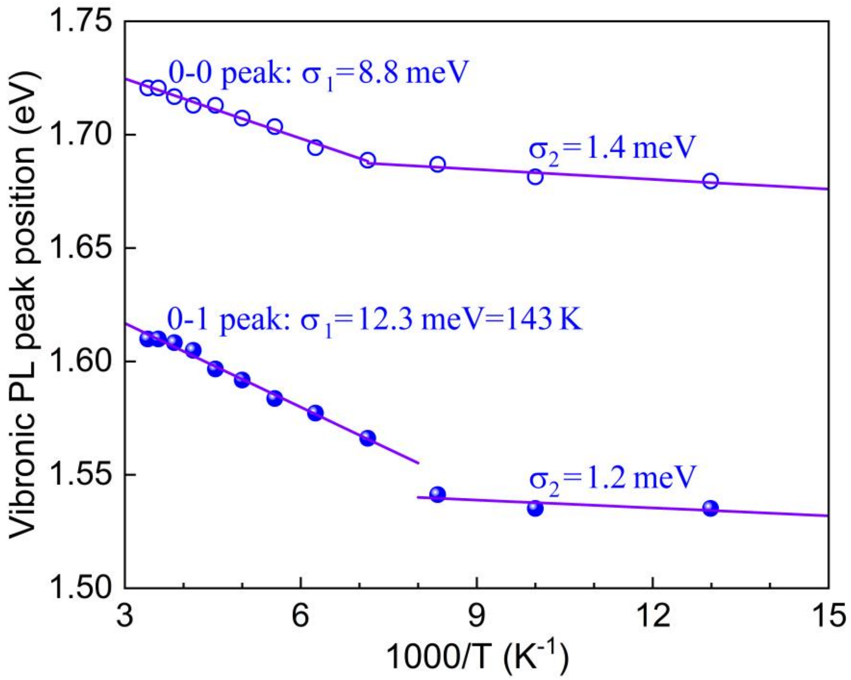

Fig. S4. Estimate of energetic disorder (σ) in IDTBT thin-film, based on an inverse temperature (1/*T*)-dependence of shift in the location of the 0-0 ($E_{0\text{-}0}$, open blue circle) and 0-1 ($E_{0\text{-}1}$, filled blue circle) vibronic PL peaks. Here, the slope of the fitting lines for the $E_{0\text{-}0}$ and $E_{0\text{-}1}$ shift vs 1/*T* gives an estimate of the degree of energetic disorder (σ) in IDTBT thin-film, following previous works (Refs. S2–S4) by treating the thermal relaxation of photogenerated excitons towards the tail states of the electronic density of states (DOS), in terms of concomitant filling of the occupied states of DOS at a mean energy of $-\sigma^2/kT$ lying below the center of the DOS. The slopes of the solid violet fitting lines in the two temperature ranges of each dataset give two different estimates of energy disorder (σ_1 and σ_2, respectively, for the 0-0 and 0-1 vibronic transition band) at the excited state. A sudden change in slope of the fitting line for $E_{0\text{-}0}$ and $E_{0\text{-}1}$ shift is seen to occur at ~140 K. Thus, we arrive at a much higher degree of energetic disorder (σ_1 = 8.8 meV from $E_{0\text{-}0}$ vs 1/*T* and 12.3 meV from $E_{0\text{-}1}$ vs 1/*T*) for a temperature range from 295 K to 140 K than that between 140 K and 77 K (σ_2 = 1.4 meV for $E_{0\text{-}0}$ and 1.2 meV for $E_{0\text{-}1}$). On average, these estimates of energy disorder would comply with the ~690 times enhancement in mobility (μ) obtained for 1D transport along IDTBT backbone relative to that of 3D transport in an IDTBT thin-film if μ is assumed to decrease with σ as a power law (Ref. S5), $\mu \propto \sigma^{-\nu}$, with the exponent ν being calculated as 3.1 ± 0.4 for IDTBT copolymer.

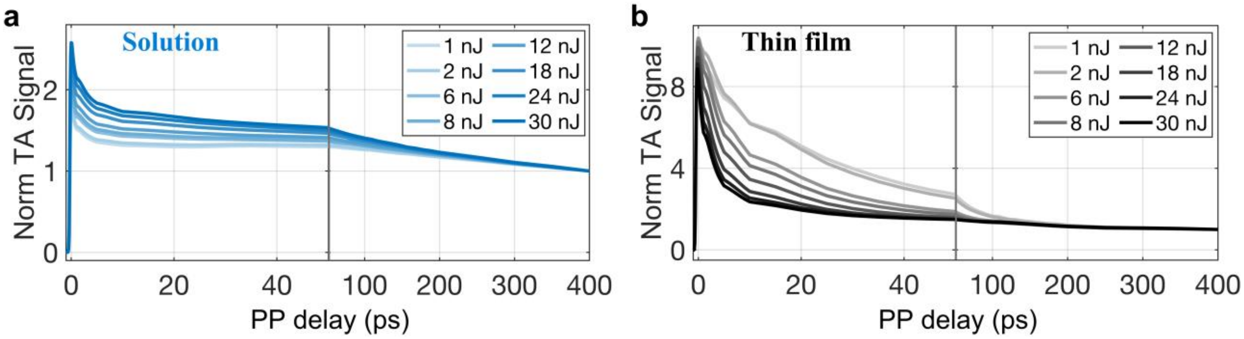

Fig. S5. 'Traditionally measured' excitation intensity-dependent transient absorption (TA) kinetics of IDTBT in its solution (**a**) and thin-film (**b**) by exciting the 0-0 transition and detecting around the maxima of ground state bleach (680 nm). All transients are recorded at room temperature and normalized to the tail signal averaged between delay time from 350 ps to 400 ps.

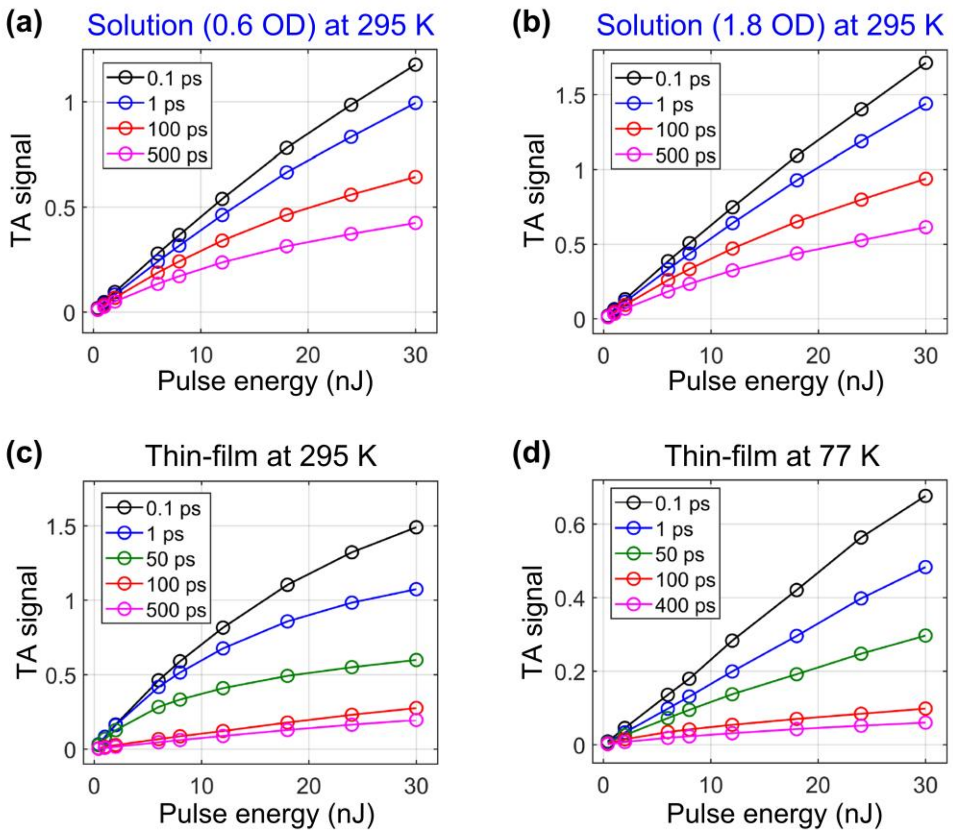

Fig. S6. (a−b) The intensity of TA signals recorded in two IDTBT solutions at various delay times, plotted as a function of pulse energy (here, 1.3 nJ pulse energy = single exciton per chain on average), and **(c−d)** the corresponding TA intensities recorded from IDTBT thin films at room temperature and 77 K. Here, the IDTBT solution samples were measured with two optical paths (OD) of 0.6 in (a) vs 1.8 in (b). For both solution samples, a linear dependence of TA signal is seen for both solutions at early time within our pump-probe range, along with a non-linear dependence at longer time due to the occurrence and increasing dominance of nonlinear exciton-exciton annihilation upon higher fluences. IDTBT thin films, on the other hand, exhibit strong nonlinear dependence of the room-temperature TA signal at all plotted delay times when the pulse energy exceeds ~10 nJ because in this case, IDTBT backbones in the solid state are close packed into an *HJ*-aggregated polymer network, in which the interchain excitonic species and ultrafast EEA kinetics dominate the TA responses. Nevertheless, recovery to a linear dependence on pulse energy was observed in IDTBT thin film at low temperatures of <140 K (e.g. 77 K in (d)) since the interchain transport and resulting EEA are significantly suppressed in this temperature range according to the findings in the main text. These plots confirm that we were measuring in a multi-particle interaction regime and strongly suggests that EEA dominates the rise time of the PP5 signal and the multi-particle kinetics in PP7 presented in the main text.

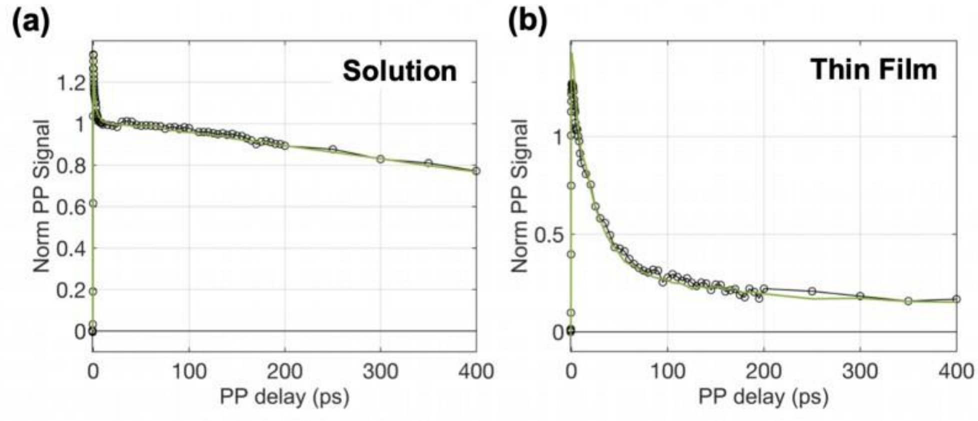

Fig. S7. Comparison of isolated room-temperature PP3 kinetic profile (green curve) with the TA profile (black circle) measured at a low pump pulse energy (here 0.4 nJ), with both signals being normalized to the tail signal at 350-400 ps. The pump fluence was sufficiently lowered so that the exciton-exciton annihilation is very unlikely and therefore, negligible contributions come from the higher order non-linear signal. Overlap of the isolated PP3 traces with the corresponding transient profile at the low pump fluence confirms successful separation of third order signal from higher order signal by applying the phase-cycling based scheme described in the main text.

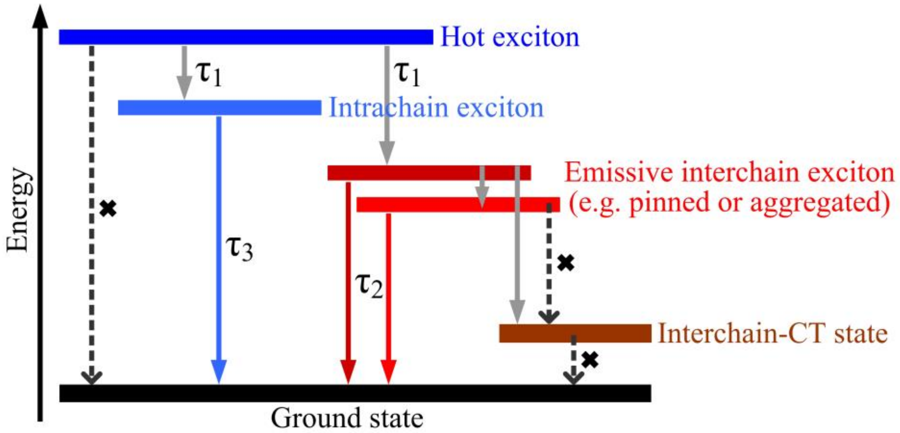

Fig. S8. Schematic illustration for the energy levels and decay feature of the three excited states of IDTBT copolymer identified from the phase-cycled TA spectroscopic measurements according to the PP3-extracted lifetimes of τ_1 (hot excitons), τ_2 (interchain excitons) and τ_3 (intrachain excitons). Here, the non-emissive, long-lived lower-energy interchain charge-transfer (CT) states, which are beyond the probe window, are also shown for completeness for the IDTBT thin film. The black dashed arrows denote unlikely decay pathways of the photogenerated excited states of IDTBT. The energy of the emissive interchain singlet excitons in IDTBT copolymer thin-film is shown to be centered around the 0-1 PL peak at ~1.6 eV in IDTBT thin film but with an extremely broad width of FWHM=0.65 eV (see Ref. 39 cited in the main text), meaning that our TA setup could detect them if they are photogenerated in IDTBT thin film. Branching of intrachain- and interchain- excitons from the photogenerated hot excitons in IDTBT thin-film occurs during the ultrafast cooling process of hot excitons. This seems to exclude the possibility of exciton transfer (from an intrachain exciton to an interchain exciton) since the chain-chain coupling sites had already been occupied by the interchain excitons in the process of earlier exciton branching.

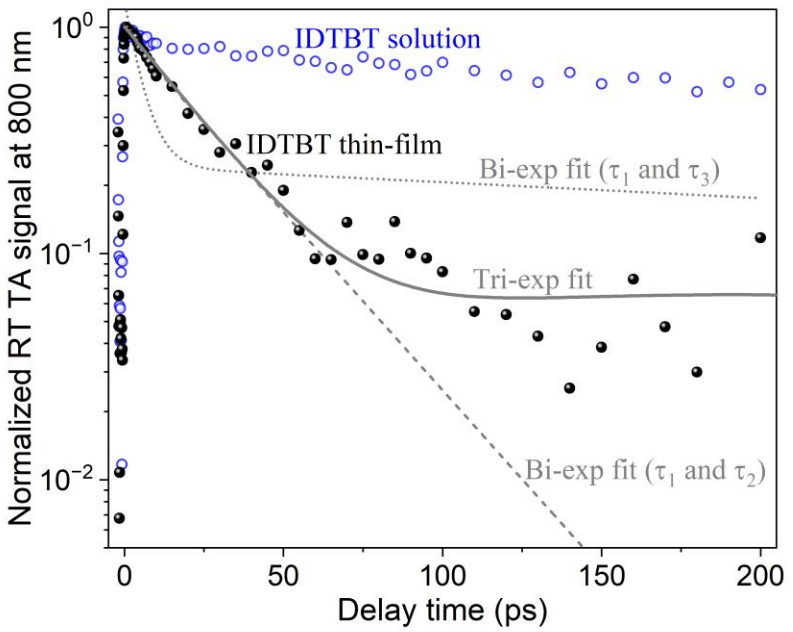

Fig. S9. Room-temperature TA spectroscopy pumped at 1 nJ pulse energy and probed at 800 nm in IDTBT solution (blue open datapoints) and IDTBT thin-film (black filled datapoints). The three fitting curves were compared for the thin-film datapoints, using two- and three-exponential decay fits with the same exciton lifetimes presented in the main text. For the solution, we found that the 800 nm-probed TA signal has the same one-particle decay with the corresponding solution PP3 signal extracted in the main text for the probe at 680 nm due to the dominant fluorescence of intrachain excitons. In the case of IDTBT thin-film, the interchain excitons (τ_2) are shown to be required for a satisfactory fit (the solid fit curve) and thus are emissive at 800 nm. Also, here we estimated a 4× PLQE of the τ_2=27.6 ps emissive interchain excitons for NIR emission (e.g., at 800 nm) than the PLQE of the intrachain excitons in IDTBT thin-film, via comparing the ration of amplitude of the interchain excitons and intrachain excitons between the three-exponential fit of 800 nm-collected TA signal (≈13.6:1) here with the ratio (≈3.4:1) of the isolated PP3 signal probed at 680 nm.

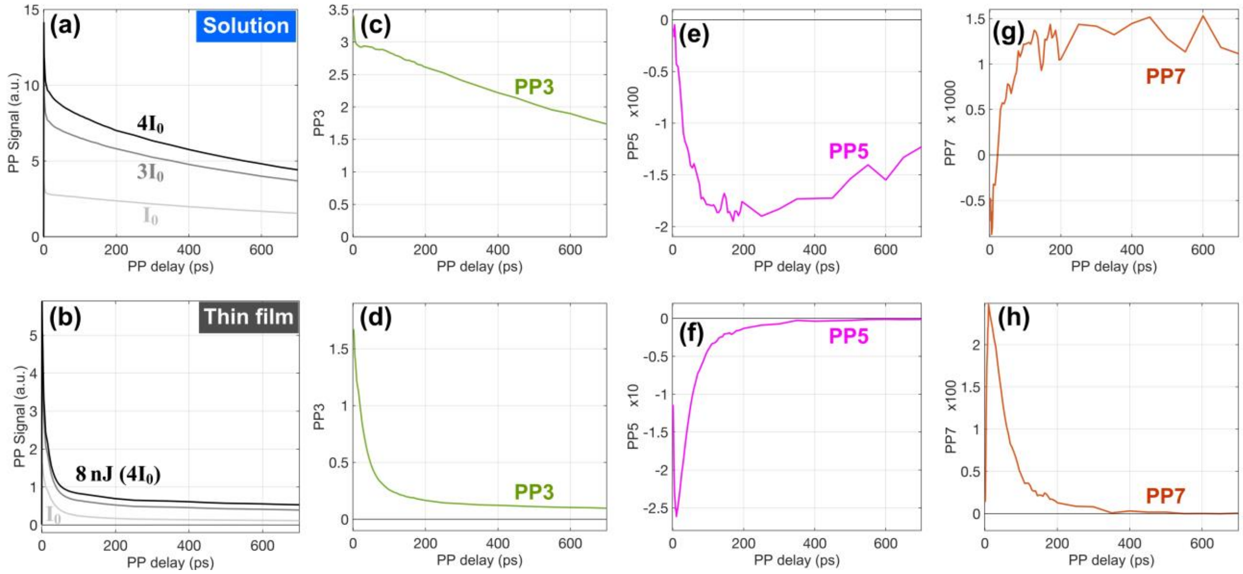

Fig. S10. Single-, two- and three-exciton dynamics from the phase-cycled TA spectroscopy in IDTBT solution (top panels) and IDTBT thin-film (bottom panels). The room-temperature kinetic TA profiles upto 700 ps measured at three prescribed pump pulse energies: I_0 = 6 nJ, $3I_0$ = 18 nJ and $4I_0$ = 24 nJ for solution (**a**), as well as I_0 = 2 nJ, $3I_0$ = 6 nJ and $4I_0$ = 8 nJ for thin film (**b**). The isolated third-order (PP3) (**c**−**d**), fifth-order (PP5) (**e**−**f**) and seventh-order (PP7) (**g**−**h**) nonlinear kinetic signals are shown by green, magenta and orange curves, respectively.

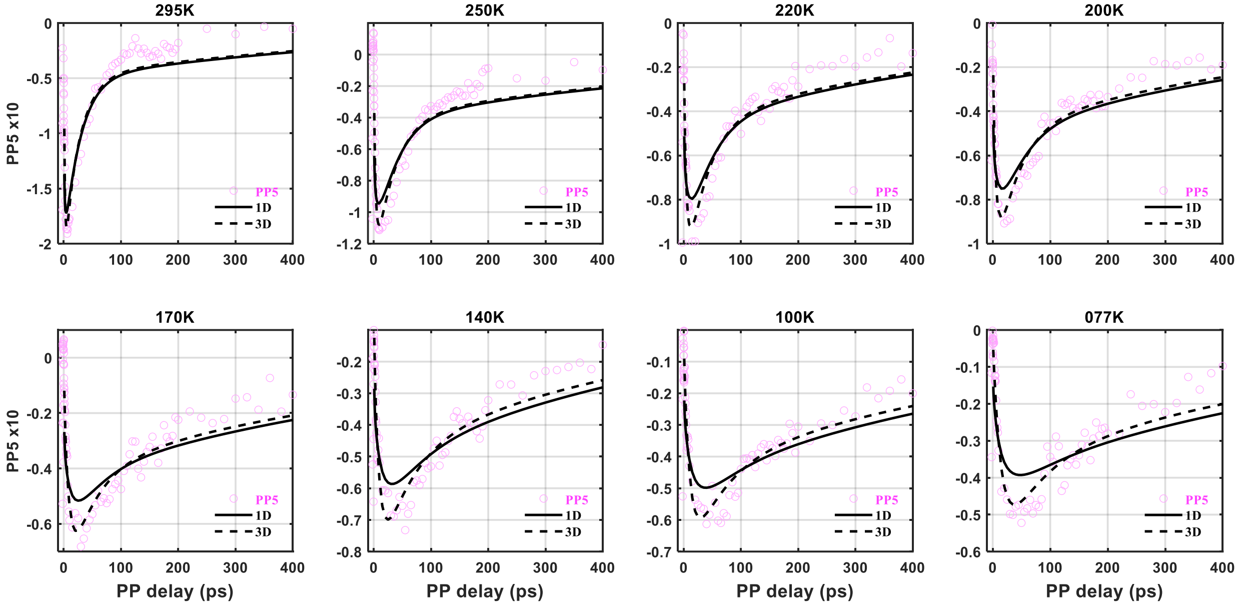

Fig. S11. Isolated fifth-order nonlinear signals (PP5, magenta circle) from the phase-cycled TA spectroscopy and fits with 1D (solid black line) and 3D (dotted black line) diffusion model for IDTBT thin-film, ranging from room-temperatures (295 K) to cryogenic temperatures (77 K).

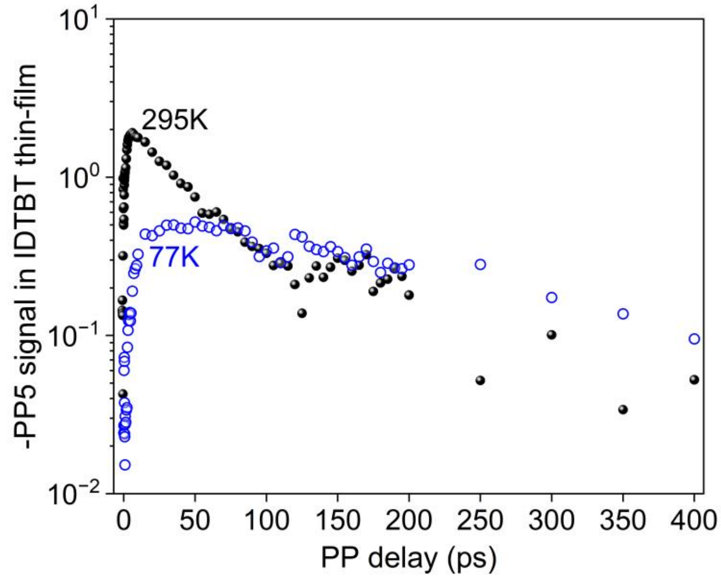

Fig. S12. Comparison of peak-normalized thin film PP5 kinetic profiles recorded at 295K (black) and 77K (blue).

Table S1. Temperature-dependent exciton decay and migration parameters obtained by fitting the isolated PP3 and PP5 kinetics with a diffusion-limited kinetic model in IDTBT thin-film.

Sample	Temperature	***PP3 fit***	***PP5 fit***		
		τ_{avg} (ps)	3D k_A or 1D k_{fit}	*diffusion coeff.* (10^{-2} cm^2 s^{-1})	*diffusion length* (nm)
IDTBT thin film	295 K	125	0.478 ps^{-1} [3D]	7.48	74.9 (±3)
	250 K	154	0.237 ps^{-1} [3D]	3.71	58.6 (±3)
	220 K	181	0.184 ps^{-1} [3D]	2.87	55.9 (±3)
	200 K	195	0.136 ps^{-1} [3D]	2.13	53.3 (±3)
	170 K	222	0.114 ps^{-1} [3D]	1.79	48.8 (±5)
	140 K	257	0.106 ps^{-1} [3D]	1.65	50.4 (±3)
	100 K	277	0.088 ps^{-1} [3D]	1.39	48.0 (±3)
	77 K	320	0.064 ps^{-1} [3D]	1.00	43.7 (±3)

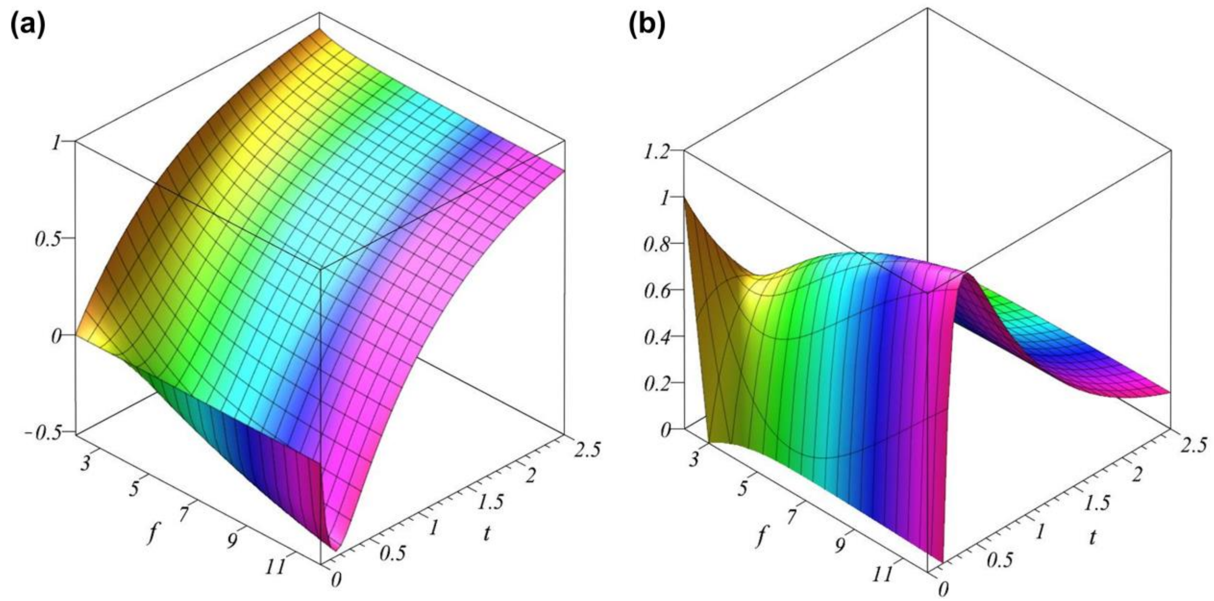

Fig. S13. (**a**) 3D function plot of the last term $\mathcal{D}_3(f, t) = 1 - \frac{2f-3}{f-1}e^{-k_A t} + \frac{f-2}{f-1}e^{-f k_A t}$ in the main text Equation 4 and (**b**) the first derivative of $\mathcal{D}_3$ with respect to the (normalized) delay time t at various values of the f parameter ranging from $f = 2$ to $f = 12$.

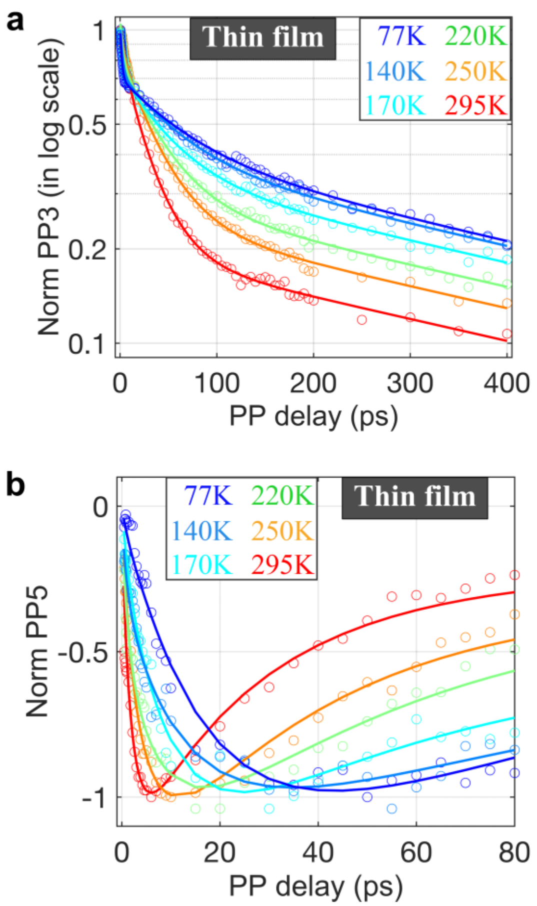

Fig. S14. Isolated single- and two-exciton dynamic results in IDTBT thin film at different temperatures. Temperature-dependent PP3 (**a**) and PP5 (**b**) nonlinear kinetic signals and the corresponding fits (solid line). The peak-normalized PP3 kinetic traces in (**a**) are fitted using a tri-exponential decay function by fixing the longest time constant at 620 ps. The PP5 signals in (**b**) are fitted with Eq. **2** by imposing 3D and 1D diffusion model, respectively, whilst using the pre-fitted dynamic parameters of the one-exciton PP3 signal presented in (**a**).

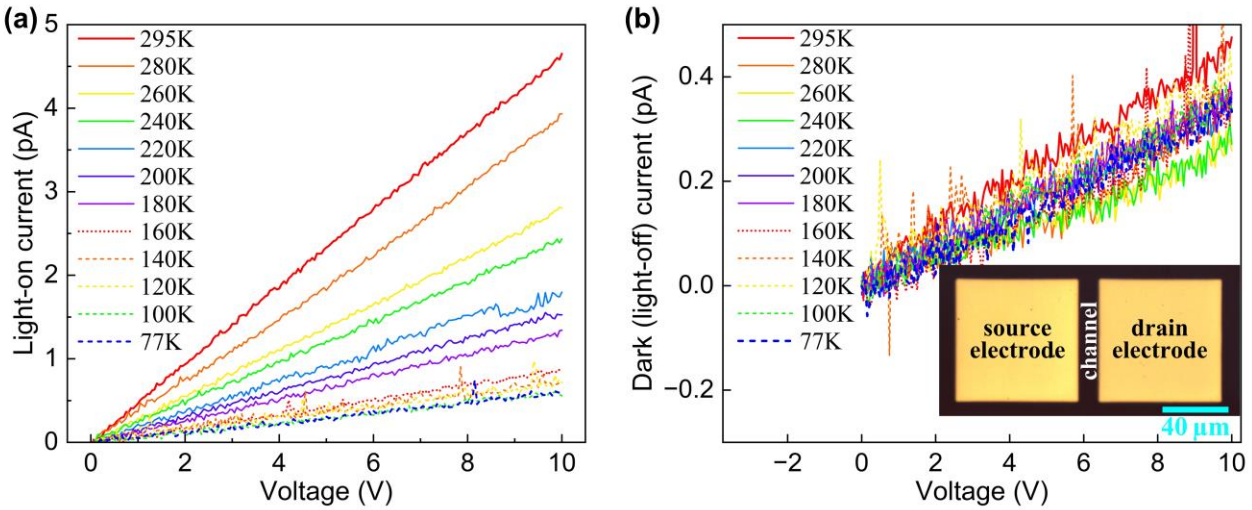

Fig. S15. Photocurrent response measures the source-drain current flow across an IDTBT thin-film transport micro-channel at different temperatures, when the continuous wave (cw) laser illumination (wavelength: 656.1 nm) on the IDTBT channel was turned on (**a**) and off (**b**). Here, the photocurrent signal was plotted against the applied source-to-drain voltage. The insert in (**b**) shows an optical image of an IDTBT thin-film transport channel and the deposited square source and drain gold electrode; the scale bar: 40 μm.

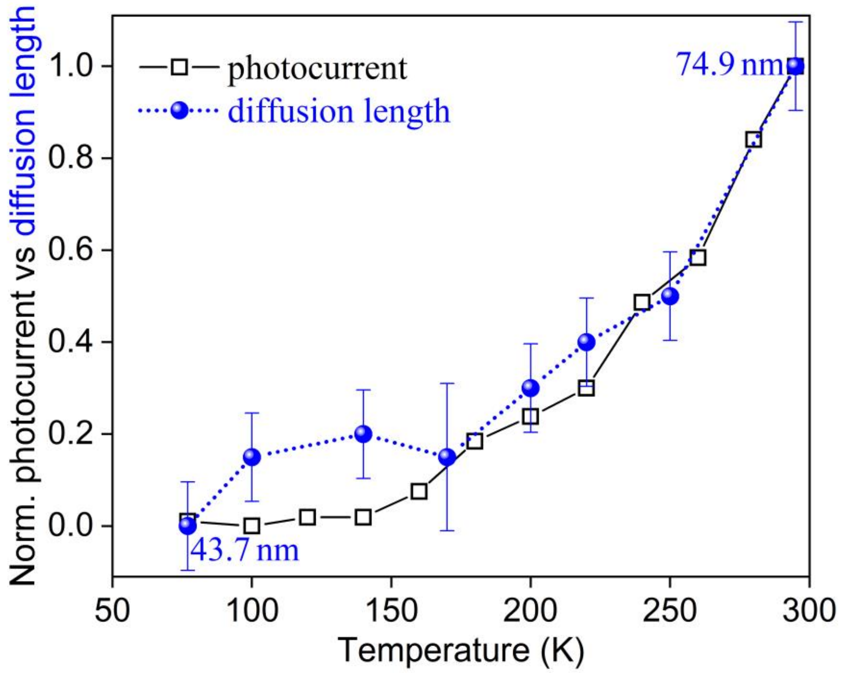

Fig. S16. Comparison of the (normalized) photocurrent signal and the thin-film exciton diffusion length at various temperatures. Both thin-film photocurrent data and PP5-fitted diffusion lengths are normalized as unity at the maximum and zero at the minimum in the measured temperature range.

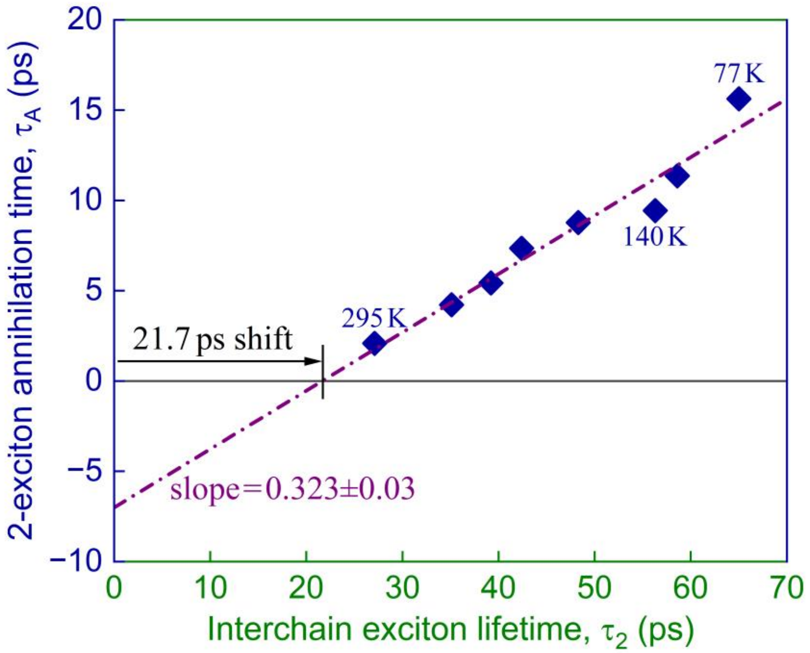

Fig. S17. A linear fit (dash-dotted purple line) of the two-exciton annihilation time ($\tau_A = 1/k_A$, filled diamonds) vs the interchain exciton lifetime (τ_2) in an IDTBT thin-film, with both data being extracted from thin-film PP5 traces at temperatures ranging from 295 K to 77 K. It is shown that the slope of the fitting line is ≈1/3, implying that the effective lifetime of interchain excitons are tripled in terms of allowing them to encounter and separate several times (i.e., ≪100% per-encounter annihilation probability). Also, the liner fit is right-shifted from the origin along the τ_2 axis, which can be ascribed to a decrease (or an equivalent increase) in the annihilation time (or rate) by $\tau_{A,intra} = 21.7 \pm 4.7$ ps (or 0.046 ± 0.013 ps^{-1}) arising from the annihilation incidents occurring between intrachain exciton pairs. By comparing this intrachain exciton EEA rate with the PP5-extracted overall EEA rates for the thin film (e.g. 0.478 ps^{-1} at room temperature) in Table S1, it can be seen that the interchain exciton annihilation events are the dominant contributor to the annihilation-limited excitation transport phenomena observed in IDTBT thin films.

SECTION II. Two-component PP5 Analysis

In order to include the extra two-particle kinetic contribution arising from long-lived charge-like quenching of excitons, we have extended the original one-component fitting method of **Eq. 2** in the main text to two components, and used this approach for PP5 analysis within this SI Section. These additional analyses and results indicate that long-lived charge transfer/separated excited states mainly contribute to the tail decay of PP5 signal and only induce a minor alternation (<5%) in the extraction of the EEA rates and exciton diffusion lengths resulting from much faster PP5 rise time.

We describe the PP5 response via two components:

$$PP5 = \alpha(1 - \exp(-\boldsymbol{k_A} t)) \sum A_i \exp\left(-\frac{t}{\boldsymbol{\tau_i}}\right) + \beta(1 - \exp(-\boldsymbol{k_B} t)) \sum A_j \exp\left(-\frac{t}{\boldsymbol{\tau_j}}\right) \quad \textbf{[S1]}$$

where *i* and *j* are the index of the photogenerated exciton species with a lifetime of τ_i (or τ_j), while A_i (or A_j) represents the relative population of the exciton species *i* (or *j*) that undergo exciton-exciton annihilation at a rate of k_A or polaron-exciton quenching at a rate of k_B. Here, α and β are the contribution of exciton-exciton annihilation and polaron-exciton quenching, respectively, toward PP5 signal.

Our original one-component fits in Fig. 2 and Fig. S11 of the isolated thin-film PP5 signals do not fit well in the PP5 tail (>100-400 ps range), and there is also a non-zero tail in the corresponding PP3 profiles. These observations suggest the additional 2nd component in **Eq. S1** to improve the fit of the longer-time PP5 signals by separating the hole/electron quenching dynamics from the original k_A values.

Moreover, the migration velocity of certain exciton species varies with temperature, affecting both the rise time and PP5 amplitude. As shown in Fig. S12, the PP5 rise time constant increases at lower temperatures, followed by a slower decay. This behavior possibly results from the reduced migration velocity of short-lifetime exciton species, leading to fewer and slower two-particle interaction dynamics.

For the isolated two-particle PP5 decays, we found that in IDTBT thin-film the isolated PP5 kinetic profiles at different temperatures, within the noise, converge for times >80 ps (see **Fig. S12**), along with significant temperature dependency of short-lifetime species in terms of its contribution in PP3 and PP5 (Fig. S14). This result suggests that the PP5 tail is dominated by (a minor phase of) temperature-independent excited states (such as the intrachain excitons whose

lifetime (τ_3) remains nearly constant at ~620 ps in Fig. S14a, and/or exciton quenching arising from long-lived charged states following exciton branching and on-chain charge transfer of relaxing hot excitons). Meanwhile, the strong temperature dependence of the early-time PP5 profile and rise time in IDTBT thin-film shows that the one-component PP5 fitting extracted k_A values are largely determined by thermally activated relative short-lived excitonic species such as the emissive interchain excitons.

In **Fig. S18**, and **Table S2**, we compare the fitting results of room-temperature IDTBT thin-film PP5 signal, using one-component (one-comp., **Eq. 2** in the main text) and two-component (two-comp., **Eq. S1**) PP5 fitting equations. For the as-shown 5th, 6th and 7th fit, we added a 5%, 20% and 100% population of long-lived charge-like states (relative to the total amplitude of the three exciton species listed in main text Table 1) into the 2nd component in **Eq. S1**. Based on these new PP5 fits, it can be seen that including the 2nd component (for describing the exciton quenching dynamics of long-lived charge-like excited states) induced only small alterations (by 3% and 5%, respectively, for 5% and 100% charge-like population that we added in the 2nd component in **Eq. S1**) to the original one-component fitting results (k_A and exciton diffusion lengths) in IDTBT thin film, though this 2nd addition in **Eq. S1** has greatly improved the fit of the tail of the two-particle PP5 profile. These observations infer that the quenching influence of long-lived charge-like states is insignificant when analyzing the exciton diffusivity in IDTBT from the isolated PP5 signals.

Note that the 4th PP5 fit with two-component approach is not very realistic since it gives an extremely large value of k_B value than k_A, which contradicts our expectation based on the 1st fit using **Eq. 2**. In more detail, the one-component term fit (fit #1) at room temperature and other temperatures generally has a larger fitted value than the isolated PP5 signals in the tail range (see the right panel in Fig. 2b and Fig. S11), suggesting that the one-component fitted k_A in Table 1 and Table S1 should be larger than the real exciton quenching rate by long-lived charge-like excited states. Also, since hot excitons relax their extra energy via on-chain charge-transfer migration due to HOMO-LUMO orbital overlap and intermolecular electronic coupling, we expect that the dynamics of on-chain charge-transfer excited states on PP5 kinetics has already been included within the dynamics of hot excitons (τ_1).

Table S2. Comparison of one-component (Eq. **2** main text) and two-component (Eq. **S1**) two-particle kinetic fitting results for the room-temperature thin film PP5 data. τ_i, τ_j are the lifetimes of thin-film PP3 kinetics listed in main text Table 1, and A_1=3.238, A_2=9.215, A_3=2.771.

**PP5 Fitting Method**	**α**	k_A (ps^{-1})	**β**	k_B (ps^{-1})
1st : one-comp fit (τ_1+ τ_2+ τ_3)	1.763	0.478	-	-
2nd : one-comp fit (τ_2)	3.212	0.325	-	-
3rd : two-comp fit (τ_2; τ_1+ τ_3)	2.545	0.470	1.183	0.081
4th : two-comp fit (τ_1+ τ_2; τ_3)	2.466	0.257	1.189	49.851
5th : two-comp fit (τ_2; τ_1+ τ_3+5%CT)	2.578	0.466	0.874	0.082
6th : two-comp fit (τ_2; τ_1+τ_3+20%CT)	2.619	0.461	0.487	0.084
7th : two-comp fit (τ_2; τ_1+τ_3+100%CT)	2.643	0.459	0.258	0.085

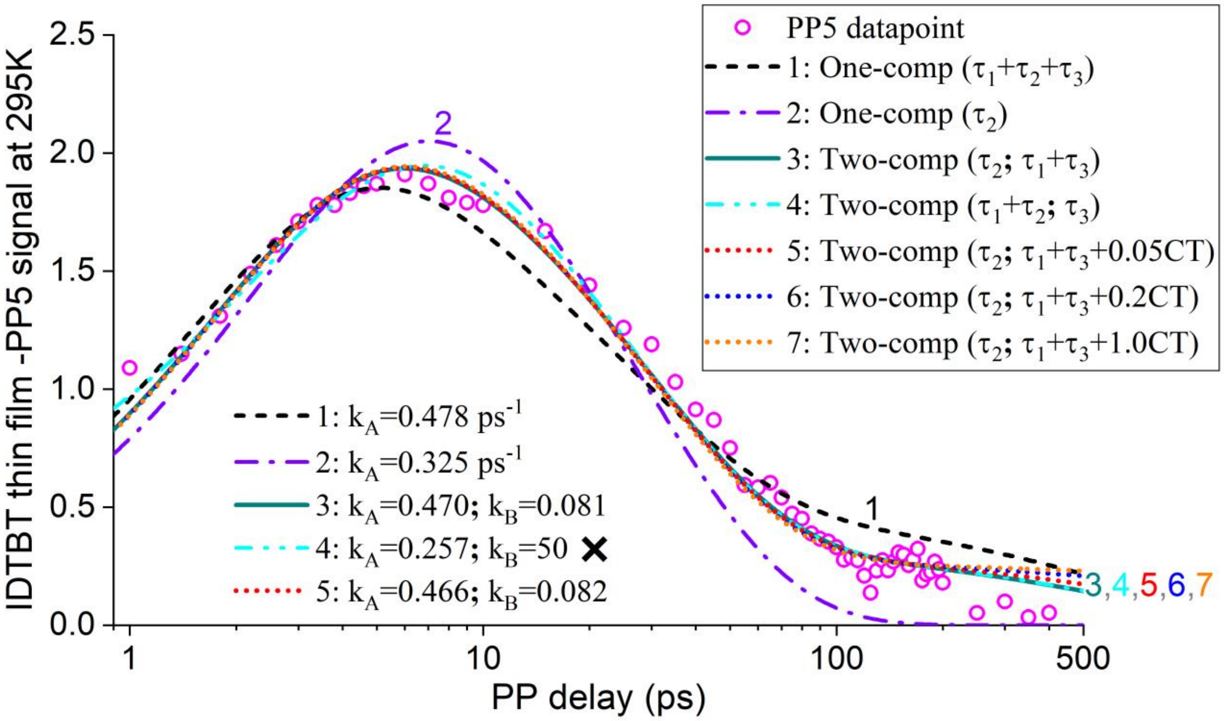

Fig. S18. Comparison of the fitting results of extracted IDTBT thin-film PP5 kinetic profile at room temperature, using one-component (one-comp. of Eq. 2 in the main text) and two-component (Eq. S1) PP5 fitting equations. For all fits plotted here, the same lifetimes and amplitudes of the three excited states in Table 1 are used, with τ_1, τ_2 and τ_3 denoting the decay constant of hot excitons, emissive interchain excitons, and intrachain excitons, whilst letting free a coefficient (α and β in Eq. S1) for each two-particle decay mechanism (k_A for exciton-exciton annihilation, k_B for polaron quenching of excitons). For the as-shown 5th, 6th and 7th fitting curve, a 5%, 20% and 100% population of long-lived charge-transfer (CT) states (lifetime is assumed to be 1 microsecond) was added to show the hole-quenching effect of excitons on the extracted k_A.

SECTION III. Photocurrent of PCBM/IDTBT blend thin film

We have carried out additional photocurrent measurements in a PCBM/IDTBT blend thin film and compared the blend photocurrent results with these in IDTBT thin film. The results are presented in this SI Section. **Fig. S19** shows a direct comparison of the inverse-temperature dependence of thin-film photocurrent of IDTBT copolymer vs IDTBT/PCBM donor-acceptor blend (50%:50% weight ratio). Both films were fabricated to enable the same absorption at 656.1 nm illumination. It is evident that the photocurrent in both films follows very similar temperature dependence over the measured temperature range, indicating that both cases are dominated by the same thermally activated transport paths through the interconnected network of IDTBT chains. This strongly indicates the charge-transfer states in both materials systems are localised (*i.e.*, interchain charge-transfer/separated excited states of IDTBT are not very mobile at the measured temperatures), with a minor population in IDTBT thin-film relative to the photogenerated excitonic singlets (see also **Fig. S20**). The slightly higher energy barrier (E_a = 69.1 meV) in the blend than in IDTBT thin-film (E_a = 57.4 meV) can result from efficient charge separation of the so-called most difficult or deeply trapped excitons located at original difficult transport sites (e.g., chain kinks and chain ends), mediated by the surrounding PCBM acceptor molecules and their molecular network.

Regarding the photocurrent measurement used, we divide the response into charge generation and charge collection. On-chain short-time transport is important for charge generation at the donor-acceptor interface where short-range charge transport is needed to separate charges and avoid recombination; photocurrent collection necessitates charges to move to longer distances (*via* interchain hopping) in order to reach the current contacts. As a result, hot excitons (such as nonradiative on-chain CT states) of IDTBT and radiative intrachain excitons (τ_3) are more easily quenched by charge-like excited states than the (pinned) interchain excitons (τ_2), which is likely the case for PCBM/IDTBT blend thin-film where PCBM molecules accommodate and transport separated charges migrating from conjugated IDTBT chain segments.

In **Fig. S20**, we compare the room-temperature photocurrent signals collected in the IDTBT thin-film and the IDTBT/PCBM donor-acceptor blend thin-film, showing a ~one-third increase in the photocurrent response in the blend film. This ~1/3 increase in the procurement collected in IDTBT/PCBM blend thin-film than in IDTBT thin-film is relatively small, compared with the case of most conjugated polymers which, when blended with a sufficient fraction of PCBM in a blend thin-film, generally give 2~3 orders of magnitude enhancement in photoconductivity (see references such as Soci C. et al. “Photoconductivity of a Low-Bandgap Conjugated Polymer”. *Adv. Funct. Mater.* 2007, 17, 632–636).

In the presence of PCBM molecules as an electron acceptor, the generated hot excitons of IDTBT would be separated into charges in the form of localised polarons, rather than going through energetic relaxation along the backbone in IDTBT thin-film; these separated charges are

then long-range transported in the PCBM network. Also, the most deeply trapped excitons of IDTBT, such as those located at difficult transport sites in the IDTBT chain network, could be effectively separated at the PCBM/IDTBT interface and then long-range transported to the current contacts, enabling extra enhancement in the photocurrent in the blend film.

So we could arrive at:

$$\frac{1}{3} = \frac{A_1 + A_{inter-CT} + A_{diff}}{A_2 + A_3 - A_{diff}} = \frac{21\% + A_{inter-CT} + A_{diff}}{61\% + 18\% - A_{diff}} \qquad \text{[S2]}$$

where A_1, A_2 and A_3 denote the amplitude of hot excitons, emissive interchain excitons, and intrachain excitons in IDTBT thin film, respectively; $A_{inter\text{-}CT}$ represent the amplitude of long-lived, non-emissive interchain charge-transfer/separated excited states in IDTBT thin film, and A_{diff} the amplitude of some deeply trapped excitons located at difficult transport sites that could be efficiently charge-separated by PCBM acceptor molecules in the blend. **Eq. S2** yields: $3A_{inter-CT} + 4A_{diff} = 16\%$ which helps us to estimate $A_{inter\text{-}CT}$ < ~5% in order to maintain a positive value of A_{diff} in the photoexcited IDTBT thin-film.

It can be seen that the relative population of photogenerated long-lived charge-transfer excited states in IDTBT thin-film is not very significant, compared to the total amplitude of other singlet excitons detected with the phase-cycled TA spectroscopic measurements in this work.

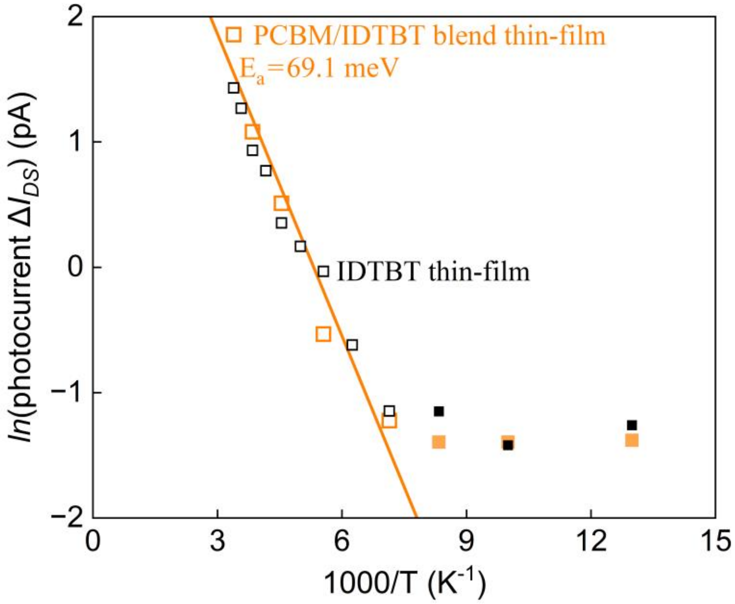

Fig. S19. Comparison of the inverse temperature (1/T)-dependence of photocurrent signals collected (at a source-drain voltage of 10 V) in the IDTBT/PCBM (50%:50% weight ratio) donor-acceptor blend thin-film (brown) and in the IDTBT thin-film (black).

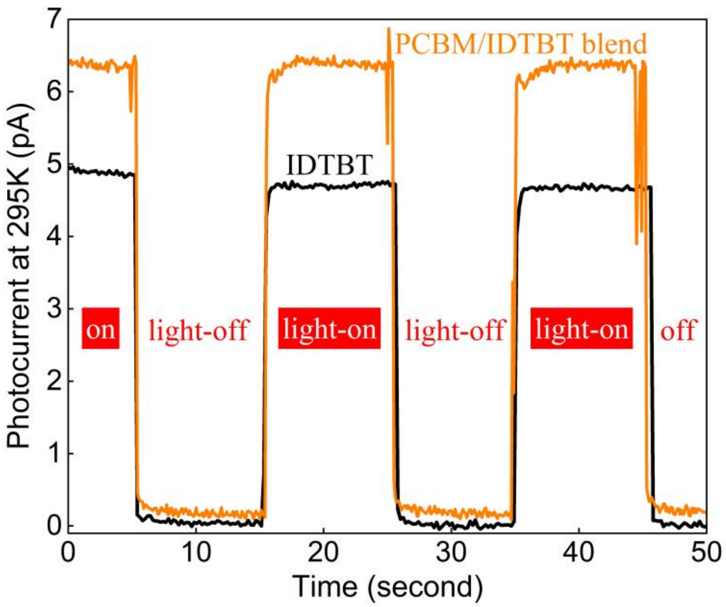

Fig. S20. Time sequence of room-temperature photocurrent signal acquired with a source-to-drain voltage of 10 V when the illumination on the PCBM/IDTBT blend thin-film (brown) or IDTBT thin-film (black) transport channel was turned on and off, showing ~one-third increase in the photocurrent in the blend film.

<u>SECTION IV</u>. List of Materials and Sources used in Fig. 3 in the main text, by material

A. Fig. 3a: *Exciton diffusion coefficients* in continuous device-relevant films of a range of semiconducting conjugated polymers and high-performing molecular acceptor films.

Regio-regular poly(3-hexylthiophene), *RR-P3HT*:

Sajjad M, Ruseckas A, Samuel, A (2020) Enhancing exciton diffusion length provides new opportunities for organic photovoltaics. *Matter* 3:341–354.

Tamai Y, Ohkita H, Benten H, Ito, S (2015) Exciton diffusion in conjugated polymers: from fundamental understanding to improvement in photovoltaic conversion efficiency. *J Phys Chem Lett* 6:3417–3428.

Poly(3-hexylthiophene-co-3-dodecylthiophene), *P3HT-co-P3DDT*:

Chowdhury M, et al. (2017) Tuning crystalline ordering by annealing and additives to study its effect on exciton diffusion in a polyalkylthiophene copolymer. *Phys Chem Chem Phys* 19:12441–12451.

Poly[2,6-(4,4-bis-(2-ethylhexyl)-4Hcyclopenta[2,1-b;3,4-b0]dithiophene)-alt-4,7-(2,1,3-benzothiadi-azole)], *PCPDTBT*:

Mikhnenko OV, et al. (2012) Exciton diffusion length in narrow bandgap polymers. *Energy Environ Sci* 5:6960–6965.

Regio-random poly(3-hexylthiophene), *RRa-P3HT*:

Sajjad M, Ruseckas A, Samuel, A (2020) Enhancing exciton diffusion length provides new opportunities for organic photovoltaics. *Matter* 3:341–354.

Mikhnenko OV, et al. (2012) Exciton diffusion length in narrow bandgap polymers. *Energy Environ Sci* 5:6960–6965.

Poly(p-phenylene vinylene) (*PPV*)-based derivatives:

Markov DE, Tanase C, Blom PWM, Wildeman J (2005) Simultaneous enhancement of charge transport and exciton diffusion in poly(p-phenylene vinylene) derivatives. *Phys Rev B* 72:045217.

Penwell SB, Ginsberg LDS, Noriega R, Ginsberg NS (2017) Resolving ultrafast exciton migration in organic solids at the nanoscale. *Nat Mater* 16:1136–1141.

Shaw PE (2009) *Measurements of exciton diffusion in conjugated polymers*, PhD thesis, University of St Andrews.

Polyfluorenes:

Sajjad MT, et al. (2019) Tuning the exciton diffusion coefficient of polyfluorene based semiconducting polymers. *Phys Status Solidi RRL* 13:1800500.

Shaw PE (2009) *Measurements of exciton diffusion in conjugated polymers*, PhD thesis, University of St Andrews.

(6,6)-Phenyl C71 butyric acid methyl ester, *PCBM*:

Hedley GJ, Ruseckas A, Samuel ID (2017) Light harvesting for organic photovoltaics. *Chem Rev* 117:796–837.

1,1-dicyanomethylene-3-indanone, *IDIC*:

Chandrabose S, et al. (2019) High exciton diffusion coefficients in fused ring electron acceptor films. *J Am Chem Soc* 141:6922–6929.

Firdaus Y, et al. (2020) Long-range exciton diffusion in molecular non-fullerene acceptors. *Nat Commun* 11:5220.

BTPTT-4F, *Y6*:

Firdaus Y, et al. (2020) Long-range exciton diffusion in molecular non-fullerene acceptors. *Nat Commun* 11:5220.

B. Fig. 3b: *Thermal activation energies* of exciton hopping in continuous films of various conjugated polymers and some high-performing aggregated *n*-type polymers

Regio-regular poly(3-hexylthiophene), *RR-P3HT*:

Sirringhaus H, Tessler N, Friend RH (1998) Integrated optoelectronic devices based on conjugated polymers. *Science* 280:1741–1744.

Sirringhaus H, et al. (1999) Two-dimensional charge transport in self-organized, high-mobility conjugated polymers. *Nature* 401:685–688.

Jimison LH, Toney MF, McCulloch I, Heeney M, Salleo A (2009) Charge-transport anisotropy due to grain boundaries in directionally crystallized thin films of regioregular poly(3-hexylthiophene). *Adv Mater* 21:1568–1572.

Kline RJ, et al. (2005) Dependence of regioregular poly(3-hexylthiophene) film morphology and field-effect mobility on molecular weight. *Macromolecules* 38:3312–3319.

Poly(2,5-Bis(3-alkylthiophen-2-yl)thieno[3,2-b]thiophenes), *PBTTT*:

Zhang X, et al. (2010) In-plane liquid crystalline texture of high-performance thienothiophene copolymer thin films. *Adv Funct Mater* 20:4098–4106.

Lee MJ, et al. (2011) Anisotropy of charge transport in a uniaxially aligned and chain-extended, high-mobility, conjugated polymer semiconductor. *Adv Funct Mater* 21:932–940.

Zhao N, et al. (2009) Polaron localization at interfaces in high-mobility microcrystalline conjugated polymers. *Adv Mater* 21:3759–3763.

Wang C, et al. (2010) Microstructural origin of high mobility in high-performance poly(thieno-thiophene) thin-film transistors. *Adv Mater* 22:697–701.

Poly[N-9’’-heptadecanyl-2,7-carbazole-alt-5,5-(4’,7’-di-2-thienyl-2’,1’,3’-benzothiadiazole)], *PCDTBT*:

Beiley ZM, et al. (2011) Morphology-dependent trap formation in high performance polymer bulk heterojunction solar cells. *Adv Energy Mater* 1:954–962.

Tseng H-R, et al. (2014) High-mobility field-effect transistors fabricated with macroscopic aligned semiconducting polymers. *Adv Mater* 26:2993–2998.

Poly[[2,5-bis(2-octadecyl)-2,3,5,6-tetrahydro-3,6-diketopyrrolo[3,4-c]pyrrole-1,4-diyl]-alt-(2-octylnonyl)-2,1,3-benzotriazole], *PDPPBT*:

Schott S, et al. (2015) Charge-transport anisotropy in a uniaxially aligned diketopyrrolopyrrole-based copolymer. *Adv Mater* 27:7356–7364.

Kronemeijer AJ, et al. (2012) A selenophene-based low-bandgap donor–acceptor polymer leading to fast ambipolar logic. *Adv Mater* 24:1558–1565.

Gruber M, et al. (2015) Enabling high-mobility, ambipolar charge-transport in a DPP-benzotriazole copolymer by side-chain engineering. *Chem Sci* 6:6949–6960.

Regio-random poly(3-hexylthiophene), *RRa-P3HT*:

Muntasir T, Chaudhary S (2015) Understanding defect distributions in polythiophenes via comparison of regioregular and regiorandom species. *J Appl Phys* 118:205504.

Poly(9,9-dioctylfluorene), *PFO*:

Kreouzis T, et al. (2006) Temperature and field dependence of hole mobility in poly(9,9-dioctylfluorene). *Phys Rev B* 73:235201.

Shaw PE (2009) *Measurements of exciton diffusion in conjugated polymers*, PhD thesis, University of St Andrews.

Poly(9,9-dioctylfluoren-2,7-diyl-co-benzothiadiazole), *F8BT*:

Faria GC, deAzevedo ER, von Seggern H (2013) Molecular origin of charge traps in polyfluorene-based semiconductors. *Macromolecules* 46:7865–7873.

Poly(9,9’dioctyl-fluorene-co-bithiophene), *F8T2*:

Sirringhaus H, et al. (2000) Mobility enhancement in conjugated polymer field-effect transistors through chain alignment in a liquid-crystalline phase. *Appl Phys Lett* 77:406–409.

Hamilton MC, Martin S, Kanicki J (2004) Field-effect mobility of organic polymer thin-film transistors. *J Chem Mater* 16:4699–4704.

Poly(9,9-dioctylfluorene-co-bis-N,N’-(4-butylphenyl)-bis-N,N’-phenyl-1,4-phenylenediamine), *PFB*:

Campbell AJ, et al. (2016) Charge-carrier density independent mobility in amorphous fluorene-triarylamine copolymers. *Adv Funct Mater* 26:3720–3729.

Rawcliffe R, Bradley DDC, Campbell AJ (2004) Comparative measurements of charge transport in semiconducting polymers and their devices. *Proc SPIE* 5519:89–99.

NN2:

Xiao M, et al. (2020) Anisotropy of charge transport in a uniaxially aligned fused electron-deficient polymer processed by solution shear coating. *Adv Mater* 32:2000063.

Poly{[N,N'-bis(2-octyldodecyl)-naphthalene-1,4,5,8-bis(dicarboximide)-2,6-diyl]-alt-5,5'-(2,2'-bithiophene)}, *P(NDI2OD-T2)*:

Caironi M, et al. (2011) Very low degree of energetic disorder as the origin of high mobility in an n-channel polymer semiconductor. *Adv Funct Mater* **21**, 3371–3381.

Rivnay J, et al. (2011) Drastic control of texture in a high performance n-type polymeric semiconductor and implications for charge transport. *Macromolecules* 44:5246–5255.

Li J, et al. (2012) The influence of gate dielectrics on a high-mobility n-type conjugated polymer in organic thin-film transistors. *Appl Phys Lett* 100:033301.

SECTION V. Systematic Error Evaluation

We evaluate the systematic error associated with our pump-probe measurement by considering both the pump intensity distribution and probe population distribution as Gaussian distributions. **Table S3** lists calculated parameters at some representative critical positions based on an 8 nJ pump intensity.

Table S3. Pump-probe error parameters calculated at some critical positions.

Critical position	Distance from Pump center (μm)	Pump fluence ($pJ/\mu m^2$)	Error of pump fluence from center	Population percentage of probe within the range
Center	0	0.28	0 %	--
1σ of probe	34	0.24	-11.8 %	39.3 %
FWHM of probe	40	0.23	-15.9 %	50.0 %
2σ of probe	68	0.17	-39.3 %	86.5 %
FWHM of pump	80	0.14	-50.0 %	93.8 %

Here, the averaged pump fluence is 0.22 $pJ/\mu m^2$ over the Gaussian distribution, which is ~20% error to the maximum fluence at center. As for the errors at various critical positions, table shows that at 1σ of the probe beam, about 40% of the detected population experiences less than ~12% error of pump fluence compared to the center. When extending distance to 2σ of probe beam, the error increases and ~14% total population experience less than 60% pump fluence of the center. Also, increasing the FWHM ratio of the pump to the probe beam directly reduces the error. For a ratio of 3 (e.g., 120 μm for pump and 40 μm for probe), the error of averaged fluence decreases to 10%, and the error at 2σ decreases to 20%. The errors can be further reduced to <10% when the ratio increases to 4.5, providing a more homogeneous excitation density over probe beam region.

A lower pump fluence directly reduces the exciton density in IDTBT and exciton encounter rates, leading to a slower rise in PP5 signal. The pump fluence distribution similarly affects the measurement of I_0, $3I_0$, and $4I_0$, introducing similar center-to-edge errors. Therefore, while the phase-cycling-based TA technique remains valid, it yields a slower PP5 rise with a Gaussian beam compared to uniform excitation.

SECTION VI. Phenomenological EEA Model

Instead of fitting PP5 rise kinetics with Eq. **2** to determine the EEA rate, a general reaction equation, $\frac{dn(t)}{dt} = -\frac{1}{2}k_A n^2 - \frac{n}{\tau}$, is widely used to phenomenologically describe exciton decay and two-exciton annihilation dynamics. The integration of $dn(t)/dt$ provides the equation for TA signal:

$$TA\ signal(t) \propto \frac{n_0 e^{-\frac{t}{\tau}}}{\frac{n_0 k_A \tau}{2}\left(1-e^{-\frac{t}{\tau}}\right)+1} \quad [S3]$$

where n_0 is exciton density per molecule, τ is the decay lifetime, and k_A is the annihilation rate that is treated as a constant in 3D diffusion reactions. In Eq. **S3**, the excitation interactions involving more than two excitons are neglected, resulting in the TA signal being a combination of 3rd and 5th order nonlinear terms, with a negligible error term of higher orders $O(n^3)$.

$$TA\ signal(t, I) = PP3\ I + PP5\ I^2 + O(I^3) \quad [S4]$$

where I is pump intensity. Substituting Eq. **S4** into Eq. **S3**, combined with the exponential decay of PP3, yields an alternative form of PP5:

$$PP5(t) = \mathrm{A}e^{-\frac{t}{\tau}}\left(\frac{1}{\frac{k_A \tau n_0}{2}\left(e^{-\frac{t}{\tau}}-1\right)-1} + 1\right) \quad [S5]$$

It is not straightforward to compare the rise kinetics between PP5 from Eq. **2** in the main text and Eq. **S5** herein, we performed a qualitative comparison by generating the PP5 curves using fixed parameters. Fig. **S21** shows that the phenomenological method (Eq. S3) results in a slower rise than the method in main text (Eq. **2**). Eq. **S5** requires a larger k_A to achieve a comparable rise kinetic of Eq. **2** result. Accordingly, fitting PP5 using this phenomenological method is expected to yield a larger k_A than the corresponding values we reported based on the separated PP5 signal from our phase-cycled TA measurement.

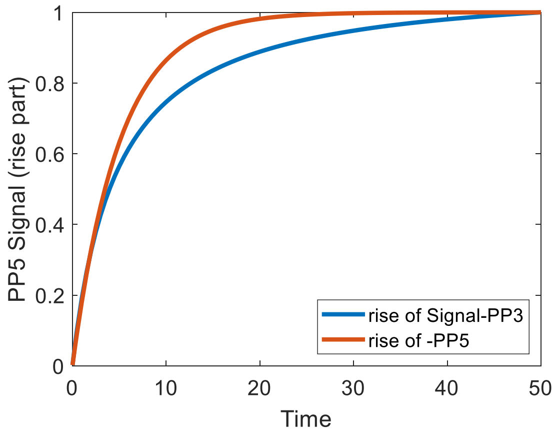

Fig. S21. Simulation of the PP5 rise component with Eq. **2** (blue) in the main text vs Eq. **S5** (orange). Here, the input parameters used are $n_0 = 1$, $k_A = 0.2\ ps^{-1}$, $\tau = 50\ ps$, and A = 1.

SI References

S1. Yamagata H, Spano FC (2012) Interplay between intrachain and interchain interactions in semiconducting polymer assemblies: The HJ-aggregate model. *J Chem Phys* 136:184901.

S2. Firdaus Y, et al. (2020) Long-range exciton diffusion in molecular non-fullerene acceptors. *Nat Commun* 11: 5220.

S3. Mikhnenko OV, Blom PWM, Nguyen T-Q (2015) Exciton diffusion in organic semiconductors. *Energy Environ Sci* 8:1867–1888.

S4. Chandrabose S. et al. (2019) High exciton diffusion coefficients in fused ring electron acceptor films. *J Am Chem Soc* 141:6922–6929.

S5. Fratini S, Nikolka M, Salleo A, Schweicher G, Sirringhaus H (2020). Charge transport in high-mobility conjugated polymers and molecular semiconductors. *Nat Mater* 19:491–502.
